# Accessing the behavior and awareness of veterinary professionals towards antimicrobials use and antimicrobial resistance in Indian district

**DOI:** 10.3389/fvets.2024.1342089

**Published:** 2024-03-11

**Authors:** Virendra S. Dhayal, Bilal Ur Rehman, Ayana Krishnan, Vijay Pal Singh

**Affiliations:** ^1^Shri Jagdishprasad Jhabarmal Tibrewala (JJT) University, Jhunjhunu, Rajasthan, India; ^2^CSIR- Institute of Genomics and Integrative Biology (CSIR-IGIB), New Delhi, India; ^3^Academy of Scientific and Innovative Research (AcSIR), Ghaziabad, India

**Keywords:** awareness, antimicrobial use (AMU), antimicrobial resistance (AMR), antimicrobial susceptibility testing (ABST), veterinary practice, Jhunjhunu

## Abstract

**Introduction:**

Antimicrobial resistance (AMR) poses a significant public health threat, and understanding the awareness and practices of healthcare professionals is crucial for its mitigation. Since the animal sector plays a key role in India’s economy, we decided to explore the understanding of Antimicrobial Use (AMU) and AMR among veterinary professionals.

**Methods:**

The study aimed to evaluate the awareness and behavior of veterinarians and para-veterinarians working in the Jhunjhunu district, Rajasthan, India, concerning AMU and AMR. Questionnaire surveys were administered to them with closed-ended questions. The data was collected and subjected to statistical analysis to derive meaningful insights. The key findings highlight notable differences in certain behavioral aspects of antibiotic prescription among the two groups.

**Results and Discussion:**

It appears that 53.8% of veterinarians as compared to 25.8% para-veterinarians do not surely inform farmers about the importance of adhering to antibiotic withdrawal periods, thereby failing to raise awareness about proper antibiotic use. Moreover, para-veterinarians (46.6%) tend to engage less in evidence-based antibiotic prescription than veterinarians (81%). Furthermore, both groups exhibit a lower frequency of advice on Antimicrobial Susceptibility Testing (ABST), essential for informed prescribing decisions. Most significantly, both groups show a tendency to prefer critically important antibiotics for prescription, raising concerns about the escalating threat of AMR. This study thus emphasizes the areas that need targeted interventions to enhance responsible antimicrobial usage and curb the growth of AMR in the region.

## 1 Introduction

Antimicrobial resistance (AMR) is a complex and multifaceted global public health concern that impacts the well-being of humans, animals, and the environment (1). Microorganisms, including bacteria, viruses, fungi, and parasites, evolve over time, rendering them unresponsive to antibiotics, fungicides, and other antimicrobials, leading to more challenging infections to treat. This phenomenon increases the risks of disease spread and contributes to a rise in illness and death (2). In 2019, AMR directly contributed to 1.27 million fatalities worldwide, a number expected to rise to a staggering 10 million by 2050 (3).

India, in particular, faces a significant battle, grappling with one of the highest rates of antimicrobial resistance globally (4). Projections indicate that by 2050, India could witness 2 million deaths attributable to AMR (5). Highlighting India’s crucial role is its status as the country with the largest livestock population worldwide, boasting an impressive 536.76 million animals. Moreover, India stands as the leading producer of milk, annually producing a substantial 221.06 million tonnes (6, 7).

The food-animal industry in India, with its immense potential for productivity, has widely employed antibiotics to enhance output (8). Various studies have observed a significant rise in antibiotic usage within the Indian population (9, 10). In 2010, India accounted for 3% of the world’s total consumption of antimicrobials in food animals, a figure anticipated to rise to 4% by 2030 (11). These statistics underscore the substantial reliance on antibiotics within the Indian food-animal sector, posing a significant risk of promoting the emergence and spread of resistance among pathogens (12). These resistance pathogens have the potential to traverse from animals to humans and the environment through a variety of routes, including direct contact, the presence of antimicrobial residues in food products (13, 14), and contamination originating from agricultural waste (4).

Apart from the increase in antimicrobial usage, India faces challenges such as a shortage of workforce and adequate knowledge dissemination to farmers (15). In many developing countries, there is often a shortage of qualified veterinarians in veterinary services (16, 17). Consequently, para-veterinarians have become crucial in delivering veterinary services, including the prescription of antibiotics for animals (18, 19). It is important to note, however, that para-veterinarians are primarily trained to perform minor services like Artificial Insemination (20). It’s critical to recognize that global regulations strictly authorize only veterinarians to prescribe antibiotics, not para-veterinarians (21, 22). Therefore, the practice of para-veterinarians prescribing antibiotics deviates from these established norms, raising legitimate concerns regarding the potential for incorrect and inappropriate antibiotic prescribing practices. Meanwhile, veterinarians following the competence criteria established by the OIE, undergo extensive training to manage livestock and prescribe antibiotics, playing a crucial role in safeguarding antibiotics and combating AMR (23, 24). Despite their pivotal role as the primary source of information for livestock producers, an awareness gap persists between veterinarians and farmers, highlighting the urgent need for enhanced communication (25).

Our previous interesting research on the Farmers of this district revealed that a significant number of them are unaware of the crucial aspects of antibiotic usage (26). Therefore, we aim to investigate factors influencing the AMR burden by examining the knowledge and communication gap, antibiotic prescription patterns, and preferences of veterinary professionals. Building upon these events, our research extends to examining the current status and prescription practices of para-veterinarians and veterinarians in India. Our present study aims to compare the differences in the behavior, and knowledge regarding AMU and AMR among veterinary professionals in Jhunjhunu district, Rajasthan, India. The outcomes of this research provide valuable insights that can enable policymakers to design and implement effective antimicrobial stewardship programs and strategies for mitigating the challenges posed by poor practices and lack of knowledge dissemination regarding antibiotics in the context of livestock production.

## 2 Materials and methods

### 2.1 Study area and population

The survey was conducted in Jhunjhunu district, located in north-eastern Rajasthan, in the north-western region of India. This district is composed of 10 sub-districts (Tehsil) and encompasses 992 villages [District (27)]. As per the 20th livestock census conducted in 2019, Rajasthan boasted a cattle and buffalo population of 13.9 and 13.7 million, respectively. Furthermore, Rajasthan accounted for 10.6% of the nation’s total milk production, emphasizing the need for health services to manage this substantial livestock population (4, 6). Conducting research in such a location provides valuable insights into understanding Antimicrobial Use (AMU) and Antimicrobial Resistance (AMR).

### 2.2 Survey design and sampling

An extensive literature review was carried out to identify the factors influencing the behavior of veterinarians and para-veterinarians regarding AMU and AMR. The questionnaire’s structure was informed by insights obtained from qualitative interviews and focus group discussions with veterinary educators and animal practitioners from various institutions.

The questionnaire itself was designed to encompass a series of close-ended questions divided into 3 sections with multiple-choice options as they are less time-consuming than descriptive questions (Annexure I). The first section comprises questions related to demographic information such as name, gender, age, and experience. The second section focuses on participants’ knowledge and awareness regarding antibiotic use, improper use, and their association with AMR, while the third section addresses behavioral aspects.

The study’s target population encompassed all veterinarians and para-veterinarians, and their participation was entirely voluntary. Participants were provided with participant information and consent forms before the dissemination of the questionnaire survey to them. We distributed the questionnaire survey via Google Forms to the participants through e-mail and other social media channels. To be eligible, the participants were required to be employed and have responsibilities related to animal treatment. In the Jhunjhunu district, we contacted many eligible participants, but only 125 professionals agreed to participate. This group included 55 Veterinarians and 70 Para-veterinarians. Some of them did not respond and a few participants responded twice to the same Google form. After eliminating duplicate responses and calculating the total responses, we obtained 110 effective responses, including 52 Veterinarians and 58 Para-veterinarians.

### 2.3 Statistical analysis

The responses were obtained in Google Forms and data was cleared and coded in Microsoft Office Excel 2021. The chi-square test was applied to the coded data to identify the relation between the Veterinarian and the Para-veterinarian responses. A *p*-value of <0.05 was considered as significant. For categories having a frequency of less than 5, the Fisher Exact test was applied. The analysis of the data was conducted using IBM SPSS Statistics 29.0.1.0. Figures of the relevant data were created using Tableau 2023.2.

## 3 Results

A total of 110 responses were obtained, including 52 (47.27%) Veterinarians and 58 (52.7%) Para-veterinarians. According to their responses, several findings were obtained.

### 3.1 Demographic information

Table 1 summarizes the demographic characteristics of participants in the veterinarian and para-veterinarian professions. The majority of the individuals in both fields are male, with 94.2 and 74.1% representation, respectively. However, the count of females is significantly higher among para-veterinarians when compared to veterinarians, indicating a potential gender preference for providing veterinary services (*p <* 0.05).

**Table 1:** Demographic characteristics of veterinarians (*n* = 52) and para-veterinarians (*n* = 58).

Parameter	Category	Veterinarian	Para-veterinarian	*p-* value
		% (*N*)	% (*N*)	
Gender	Male	94.2 (49)	74.1 (43)	<0.05
	Female	5.7 (3)	25.8 (15)	
Age	Less than 30	1.9 (1)	44.8 (26)	<0.001
	Between 31–40	51.9 (27)	50.0 (29)	ns
	Between 41–50	28.8 (15)	5.17 (3)	<0.001
	Greater than 50	17.3 (9)	0 (0)	
Experience	Less than 5	1.9 (1)	36.2 (21)	<0.001
	Between 5–10	44.2 (23)	50.0 (29)	ns
	Between 10–20	30.7 (16)	12.0 (7)	<0.001
	More than 20	23.0 (12)	1.7 (1)	
Highest qualification	BVSc & AH	73.0 (38)	1.7 (1)	<0.001
	MVSc & AH	23.0 (12)	0 (0)	
	Diploma in livestock and AH	0 (0)	94.8 (55)	
	Other diploma	0 (0)	1.7 (1)	ns
	Other	3.8 (2)	1.7 (1)	ns
Working sector	Government sector	96.1 (50)	62.0 (36)	<0.001
	Private sector	1.9 (1)	36.2 (21)	
	Other	1.9 (1)	1.7 (1)	ns

Notably, a significant portion of para-veterinarians (44.8%) begin their practice early in their career before the age of 30, while the majority of veterinarians fall within the 31 to 40 age range (51.9%). There was also a difference in the later stages of the carrier, it was observed that 28.8% of the veterinarians continue to practice between the ages of 41–50 whereas only 5.17% of the para-veterinarians practice during this age group (*p* < 0.001).

Regarding experience, veterinarians are observed to be more experienced holders as compared to para-veterinarians. Specifically, 44.2% of veterinarians and 50% of para-veterinarians have acquired less than 5 years of experience and are actively practicing. 30.7% of veterinarians have experience between 10–20 years whereas only 12% of para-veterinarians have equivalent experience (*p <* 0.001). Furthermore, in the category of professionals with over 20 years of experience, 23% are veterinarians, whereas para-veterinarians account for only 1.7% (*p <* 0.001). Qualification-wise, 73% of veterinarians pursue BVSc, while the majority of para-veterinarians (94.8%) opt for a Diploma in livestock (*p* < 0.001).

**Figure 1:**
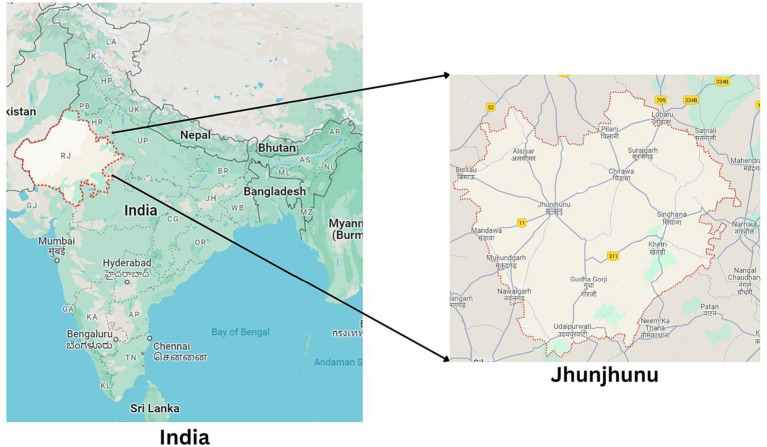
Jhunjhunu District map in Rajasthan, India.

In terms of the employment sector, 96.1% of the veterinarians and 62% of the para-veterinarians work in the government sector, reflecting a stronger inclination of veterinarians towards government service compared to para-veterinarians (*p <* 0.001). Conversely, the second major group of para-veterinarians (36.2%) is engaged in the private sector, a significantly higher proportion when compared with the mere 1.9% of veterinarians in this sector (*p* < 0.001) (see Figure 1).

### 3.2 Awareness

**Figure 2:**
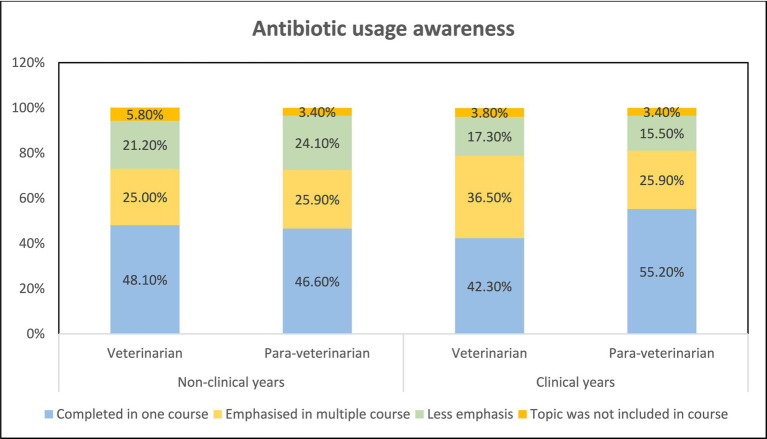
Emphasis on antibiotic usage in teaching during non-clinical and clinical years.

**Figure 3:**
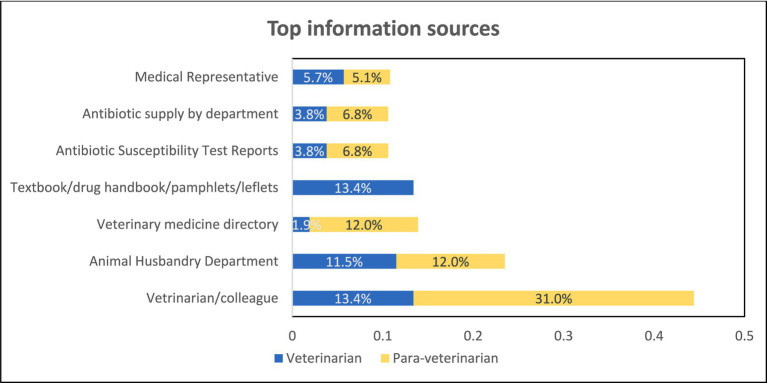
Various source of information that updates participants regarding antibiotic use.

The understanding of antibiotic usage among veterinarians and para-veterinarians during their non-clinical and clinical years of education was similar. In both groups, the majority responded that the topic was discussed only in one course, with only 25 to 37% of participants indicating that the topic was covered in multiple courses. No significant difference was observed in the responses between the two groups (Figure 2). When asked about the source of information, veterinarians, and para-veterinarians differed in their responses. Colleagues (13.4%) and textbooks (13.4%) were identified as the primary source of information regarding antimicrobial usage (AMU) among veterinarians. However, para-veterinarians reported that veterinarians/ colleagues (31%) were their main source of knowledge regarding the use of antimicrobials (Figure 3).

**Figure 4:**
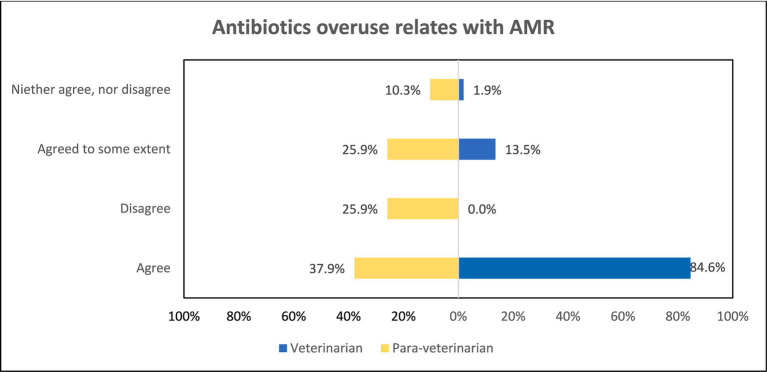
Comparison of awareness among participant groups regarding overuse of antibiotics leading to AMR.

**Figure 5:**
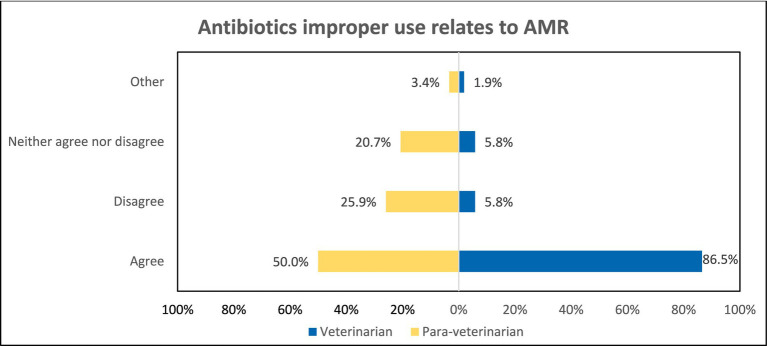
Comparison of awareness among participant groups regarding improper use of antibiotics leading to AMR.

When asked “Whether the overuse of antibiotics in animals leads to AMR,” the responses depict notable differences. Veterinarians (84.6%) seemed to be more aware as compared to para-veterinarians (37.9%) (*p* < 0.001) (Figure 4). Similarly, regarding the belief that “Improper use of antibiotics contributes to AMR,” para-veterinarians appeared less informed. Only 50% of them agree while 25.9% disagree and 20.7% neither agree nor disagree. In contrast, veterinarians exhibited greater awareness, with 86.5% in agreement (*p* < 0.001) (Figure 5). Despite these differences in awareness, both groups acknowledged the rise in antimicrobial resistance cases in their work area, with 70.7% of para-veterinarians and 63.5% of veterinarians agreeing with this statement (Table 2).

**Table 2:** Questions related to antibiotic overuse, improper use, and awareness about the increase in AMR in their work area.

Questions	Category	Veterinarian	Para-veterinarian	*p-* value
		% (*N*)	% (*N*)	
Overuse of antibiotics in animals is responsible for antibiotic resistance?	Agree	84.6 (44)	37.9 (22)	<0.001
	Disagree	0 (0)	25.9 (15)	
	Agreed to some extent	13.5 (7)	25.9 (15)	ns
	Neither agree nor disagree	1.9 (1)	10.3 (6)	ns
Improper use of antibiotics contributes to antimicrobial resistance?	Agree	86.5 (45)	50.0 (29)	<0.001
	Disagree	5.8 (3)	25.9 (15)	
	Neither agree nor disagree	5.8 (3)	20.7 (12)	
	Other	1.9 (1)	3.4 (2)	ns
There has been an increase in the number of cases of antimicrobial resistance in your work area	Agree	63.5 (33)	70.7 (41)	ns
	Disagree	19.2 (10)	17.2 (10)	
	Neither agree nor disagree	17.3 (9)	12.1 (7)	

### 3.3 Prescribing behavior

**Figure 6:**
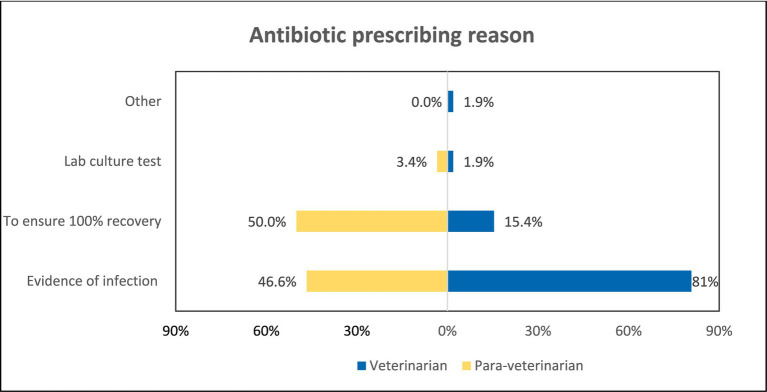
Comparison of antibiotic prescription reasons among both groups.

**Figure 7:**
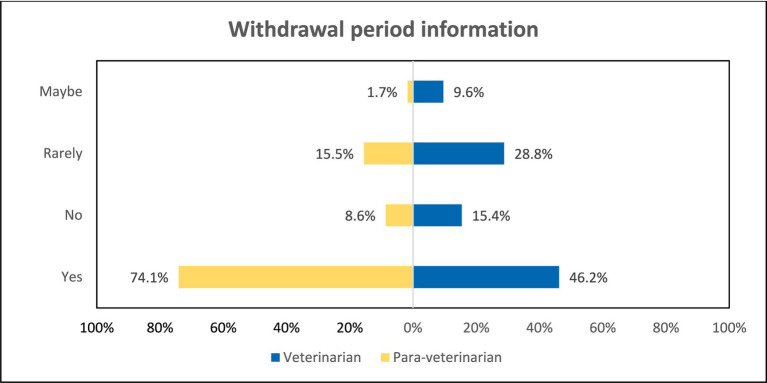
Comparison among groups regarding dissemination of withdrawal period information to farmers.

Veterinarians and para-veterinarians differ in their responses when asked about the reason for prescribing antibiotics. 80.8% of veterinarians feel that evidence of infection is the primary driver for prescribing antibiotics. In comparison, only 46.6% of para-veterinarians agree with this (*p* < 0.001). Additionally, 50% of para-veterinarians prescribe antibiotics to ensure 100% recovery of the animal, which differs significantly from the 15.4% of veterinarians who do the same (*p* < 0.001) (Figure 6). It is also observed that Veterinarians were highly likely (OR = 4.74) to prescribe antibiotics on evident infection (Table 3). Upon asking about the reading of the label before antibiotic use, both groups agreed in the same manner; 96.2 and 96.6% for Veterinarian and Para-veterinarian, respectively. Another crucial aspect of good prescription practices is conveying drug-related information to the animal owner. Regarding the communication of the information about the withdrawal period of the antibiotic before prescribing, 74.1% of the Para-veterinarians responded ‘Yes’, compared to only 46.2% of veterinarians (*p* < 0.05) (Figure 7).

**Table 3:** Questions related to antibiotic prescribing behavior by Veterinarians (*n* = 52) and Para-veterinarians (*n* = 58).

Questions	Category	Veterinarian	Para-veterinarian	*p*-value
		*N*	*N*	
What makes you prescribe antibiotics?	Evidence of infection^1^	80.8 (42)	46.6 (27)	<0.001
	To ensure 100% recovery^2^	15.4 (8)	50.0 (29)	
	Lab culture test^2^	1.9 (1)	3.4 (2)	ns
	Other^2^	1.9 (1)	0 (0)	ns
	OR = 4.74 (95% CI = 1.89, 12.73)			
Do you read the label of the antibiotic before use?	Yes^1^	96.2 (50)	96.6 (56)	ns
	Rarely^2^	3.8 (2)	3.4 (2)	
	OR = 0.89 (95% CI = 0.06, 12.74)			
Do you inform cattle farmers, about the used antibiotic and its withdrawal period?	Yes^1^	46.2 (24)	74.1 (43)	<0.05
	No^2^	15.4 (8)	8.6 (5)	ns
	Rarely^2^	28.8 (15)	15.5 (9)	
	Maybe^2^	9.6 (5)	1.7 (1)	
	OR = 0.30 (95% CI = 0.12, 0.71)			
Dose calculation would be based on?	Body weight^1^	78.8 (41)	93.1 (54)	<0.05
	By experience^2^	13.5 (7)	3.4 (2)	ns
	By guess^2^	5.8 (3)	3.4 (2)	
	Always use whole vial^2^	1.9 (1)	0 (0)	
	OR = 0.27 (95% CI = 0.06, 1.02)			
How do you measure the body weight of animal?	Experience^1^	63.4 (26)	38.9 (21)	<0.05
	Idea^2^	34.1 (14)	61.1 (33)	
	Other^2^	2.4 (1)	0 (0)	ns
	OR = 2.69 (95% CI = 1.08, 6.87)			
When do you repeat the antibiotic dose?	Only when there is no improvement^1^	67.3 (35)	86.2 (50)	<0.05
	If owner calls^2^	15.4 (8)	10.3 (6)	ns
	Other^2^	17.3 (9)	3.4 (2)	<0.05
	OR = 0.33 (95% CI = 0.11, 0.92)			
What do you generally prescribe during the repeat dose of antibiotic?	Antibiotic change^1^	38.5 (20)	60.3 (35)	<0.05
	Higher dose of previously used antibiotic^2^	11.5 (6)	20.7 (12)	ns
	Dose route change	26.9 (14)	15.5 (9)	ns
	Other^2^	23.1 (12)	3.4 (2)	<0.05
	OR = 0.41 (95% CI = 0.17, 0.94)			
In the last 1 year, how many times have you advised and asked for an antimicrobial susceptibility test?	1–5 times^1^	50 (26)	75.9 (44)	<0.05
	>20 times^2^	25 (13)	12.1 (7)	ns
	6–10 times^2^	17.3 (9)	8.6 (5)	
	16–20 times^2^	3.8 (2)	3.4 (2)	
	11–15 times^2^	3.8 (2)	0 (0)	
	OR = 0.32 (95% CI = 0.12, 0.76)			
Approx distance (in KMs) of any laboratory, that has the facility of antimicrobial susceptibility testing from your practicing area?	>20 km^1^	63.5 (33)	55.2 (32)	ns
	1–3 km^2^	13.5 (7)	20.7 (12)	
	10–15 km^2^	9.6 (5)	6.9 (4)	
	15–20 km^2^	9.6 (5)	6.9 (4)	
	4-7km^2^	3.8 (2)	8.6 (5)	
	7-10km^2^	0 (0)	1.7 (1)	
	OR = 1.40 (95% CI = 0.61, 3.26)			
Approx turnaround time for the antimicrobial susceptibility test results?	1–3 days^1^	67.3 (35)	65.5 (38)	ns
	4–5 days^2^	19.2 (10)	22.4 (13)	
	>10 days^2^	9.6 (5)	6.9 (4)	
	5–7 days^2^	1.9 (1)	3.4 (2)	
	8–10 days^2^	1.9 (1)	1.7 (1)	
	OR = 1.08 (95% CI = 0.45, 2.59)			
Is there any antibiotic that you feel comfortable prescribing?	Yes^1^	63.5 (33)	79.3 (46)	ns
	No^2^	36.5 (19)	20.7 (12)	
	OR = 0.45 (95% CI = 0.17, 1.14)			

1 Reference.

2 Categories were merged to estimate the odds ratio in reference to standard practice.

**Figure 8:**
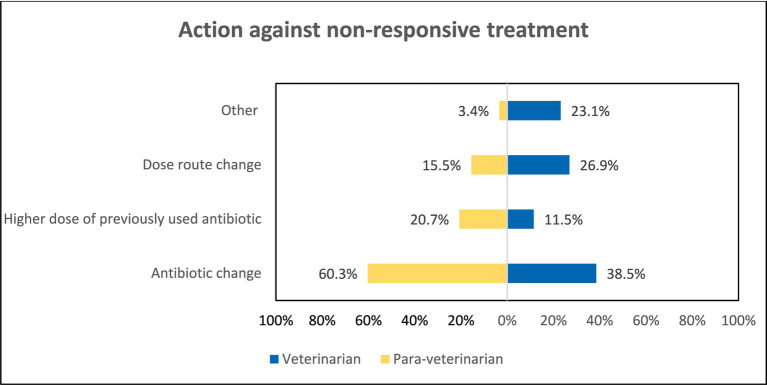
Comparison of actions of participants regarding non-responsive treatment.

There was also a significant difference observed in the methods used for Dose calculation, 93.1% of the para-veterinarians calculate dose based on Body weight while only 78.8% prefer body weight (*p* < 0.05), and 13.5% of the veterinarians prefer dose calculation by prior experience. Also, upon asking the criteria for measuring the body weight of animals 61.1% of the para-veterinarians and 34.1% of veterinarians responded that by Idea they measure body weight (*p* < 0.05). Also, Veterinarians were more likely (OR = 2.69) to use their previous experience to estimate the animal’s body weight (Table 3). When asked about the repetition of the antibiotic 86.2% of Para-veterinarians marked the reason “Only when there is no improvement” while only 67.3% of veterinarians ticked this option (*p* < 0.05) (Table 3). Furthermore, preferences for antibiotic change during repetition varied, with 60.3% of para-veterinarians and 38.5% of veterinarians favoring this practice (*p* < 0.001). Notably, 26.9% of veterinarians and 15.5% of para-veterinarians opt for a change in the route during antibiotic repetition (Figure 8).

**Figure 9:**
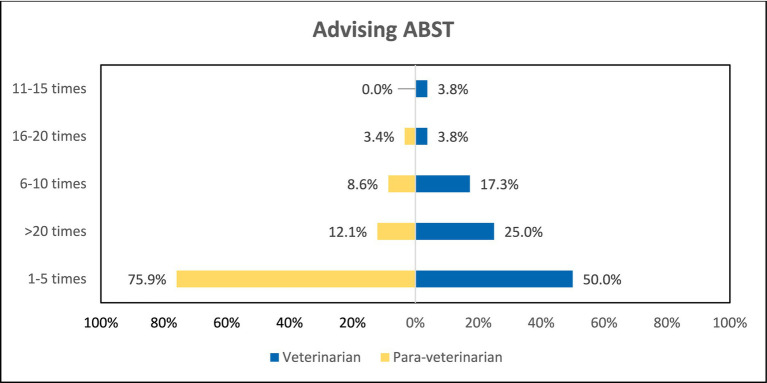
Comparison of ABST frequency among both groups.

Furthermore, participants were asked about the frequency of advised ABST in the last year. To that, 75.9% of the para-veterinarians and 50% of the veterinarians have advised only 1–5 times in the last year (*p* < 0.05). And 25% of the veterinarians and only 12.1% of the para-veterinarians advised >20 times in the last year (Figure 9). Another question response revealed that 63.5% of the veterinarians and 55.2% of the para-veterinarians agree that for the ABST the distance of the laboratory facility from the practicing area is more than 20 kilometres. Along with the distance they were also asked about the approximate turn-around time for the ABST results, majority of the participants (67.3% veterinarians and 65.5% para-veterinarians) chose 1–3 days followed by 4–5 days (19.2% veterinarians and 22.4% para-veterinarians). There was also not a significant difference in the responses between the two groups (Table 3).

**Figure 10:**
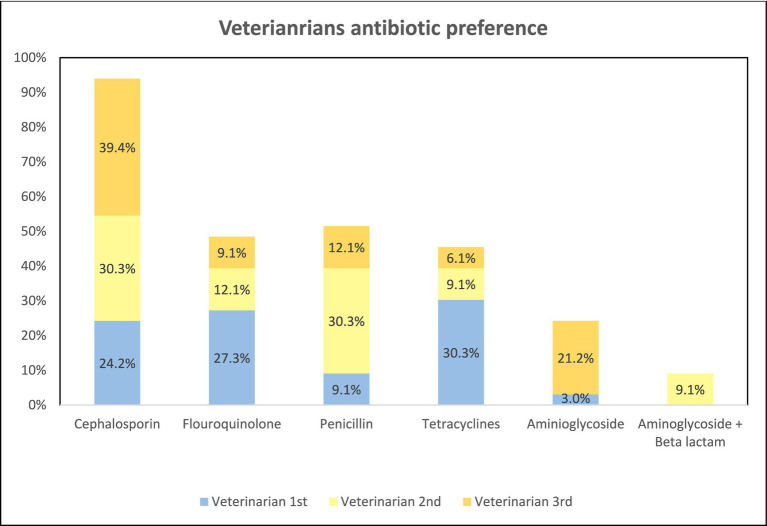
Antibiotic prescription preference by veterinarians.

When the participants were asked about the presence of any antibiotic that they felt comfortable prescribing, 63.5% of veterinarians and 79.3% of the para-veterinarians agreed with the statement (Table 3). Subsequently, we delved into exploring the top 3 preferences for antibiotics. Out of 52 veterinarians, only 33 responded about their preference for antibiotics while among 58 para-veterinarians 46 responded for the same. Among all the preferences Cephalosporins are the most preferred antibiotic among veterinarians. The 1^st^ preference of the antibiotics that veterinarians prescribe to the animal was Tetracyclines and Fluoroquinolones accounting for 30.3 and 27.3% respectively, followed by Cephalosporins accounting for 24.2%. The 2^nd^ most preferred is cephalosporins (30.3%) and Penicillin (30.3%). Among 3^rd^ most preferred antibiotics is cephalosporin (39.4%) followed by Aminoglycoside (21.2%) and Penicillin (12.1%). Within tetracyclines, fluoroquinolones, and cephalosporins classes, the most prescribed antibiotics were oxytetracycline, enrofloxacin, and ceftriaxone, respectively. However, within penicillin and aminoglycoside classes; Amoxicillin, penicillin, and gentamicin were the most chosen antibiotics (Figure 10).

**Figure 11:**
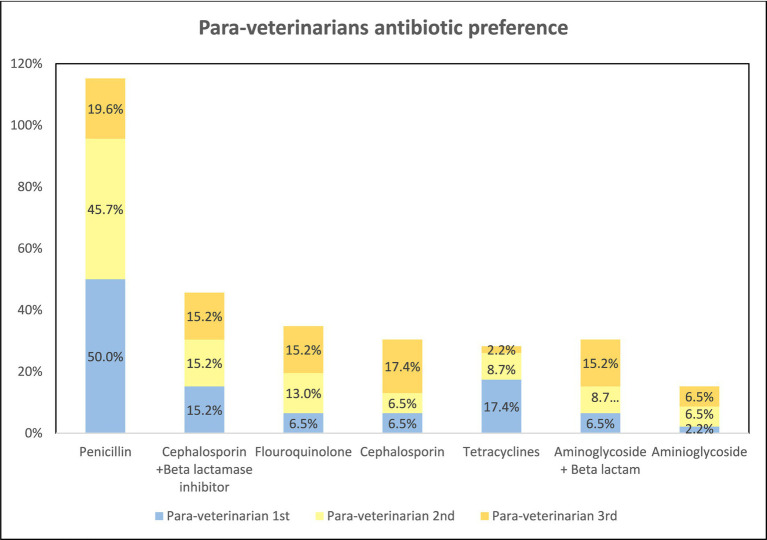
Antibiotic prescription preference by para-veterinarians.

Conversely, para-veterinarians displayed a distinct preference pattern. Penicillin emerged as their overall most preferred antibiotic, with 50% indicating it as their first choice, followed by tetracyclines (17.4%). The 2^nd^ most preferred antibiotic class is again penicillin (45.7%) followed by cephalosporin + beta-lactamase inhibitor (15.2%). The 3^rd^ most preferred antibiotic is penicillin (19.6%) followed by cephalosporin (17.4%). Among the penicillin, the Amoxicillin + cloxacillin combination was most prescribed. Within tetracyclines; oxytetracycline was preferred, within cephalosporins; ceftriaxone, and within cephalosporin + beta-lactamase inhibitor; ceftriaxone + sulbactam was among the most chosen antibiotics (Figure 11). These distinct preferences highlight variations in antibiotic prescription patterns between veterinarians and para-veterinarians.

## 4 Discussion

Understanding the awareness and behavior of veterinarians and para-veterinarians regarding antimicrobial usage could help policymakers and healthcare practitioners develop and act on strategies that mitigate Antimicrobial usage (AMU) and Antimicrobial resistance (AMR). Therefore, in this study, we examined the awareness and antibiotic prescribing behavior of veterinarians and para-veterinarians, based on the survey responses obtained. The findings indicated notable differences both the two groups in various aspects of awareness and behavior.

The study includes participants from various age groups, ensuring a range of diverse perspectives. Our study findings found that para-veterinarians showed limited awareness of the consequences of antibiotic overuse and improper leading to AMR, whereas veterinarians demonstrated greater awareness (Figures 4, 5). These results indicate that all para-veterinarians are not aware of the factors responsible for AMR, aligning with studies in India showing limited knowledge among para-veterinarians about the consequences of inappropriate antibiotic prescriptions (28, 29). Limited training programs for para-veterinarians contribute to this gap. Survey responses show para-veterinarians rely on colleagues and veterinarians for information, while veterinarians turn to textbooks and colleagues (Figure 3), consistent with previous studies (30). Another factor contributing to the participants’ limited awareness is the duration of teaching related to AMU. The survey results indicate that the emphasis on AMU topics is generally confined to a single course (Figure 2). This finding aligns with other research, which suggests that despite addressing antimicrobial stewardship there is still ample room for improvement. (31).

Most participants in both groups are aware of the increasing prevalence of AMR cases in their work area (Table 2). Unfortunately, despite this awareness, they are prescribing VCIA more frequently, raising serious concerns about AMR escalation. Effective strategies are needed to enhance veterinary professionals’ in-depth knowledge, especially para-veterinarians, and expand their understanding of antibiotic judicious use. Furthermore, establishing a national body responsible for regularly evaluating para-veterinarian performance is essential. Such initiatives are crucial in fostering awareness among veterinary professionals concerning AMU and AMR.

Transitioning from discussions about knowledge, the focus now shifts to prescription behaviors. Typically, the label/leaflet accompanying the drug dosage form contains use instructions, warnings, and other important details that should be known before administering the drug (32). It is noteworthy that the majority of both veterinarians and para-veterinarians show equal agreement when asked if they read the antibiotic label before using it, which is considered a good prescribing practice (Table 3).

The withdrawal period is a crucial concept for consumers to understand, representing the duration between the last antibiotic dose given to an animal and when antibiotic levels in the animal’s tissue fall below the maximum residual limit (33). Strict adherence minimizes the risk of antibiotic residues persisting in animal-based food products (34). However, not all veterinarians effectively convey this importance to farmers (Figure 7), potentially leading to antibiotic accumulation in products like milk and meat. Several studies also indicate that farmers often neglect or are unaware of the withdrawal period’s significance (18, 35). Hence, veterinary professionals improve farmers’ compliance with withdrawal period guidelines.

Our findings also revealed that veterinarians primarily prescribe antibiotics based on “Evidence of infection,” while para-veterinarians often prioritize ensuring 100% recovery (Figure 6). A study by Patnaik et al. in Punjab, India, analyzing the prescription pattern of both veterinarians and para-veterinarians, also found that prompt results significantly influence para-veterinarians’ prescription behavior (36). Interestingly, only a small percentage of participants rely on the Lab culture test for prescribing antibiotics (Figure 6). While prescribing antibiotics based on the infection evidence is considered a good practice, it’s important to avoid solely aiming for complete animal recovery, considering other factors like inflammation or other complications (37). Encouraging evidence-based prescription practices and providing further training and guidance to para-veterinarians on judicious antibiotic use is essential for promoting responsible antibiotic use.

Before prescribing antibiotics to animals, it is crucial to know the resistance pattern exhibited by specific antibiotics against the isolated microorganism, which can be acquired through Antimicrobial Susceptibility Testing (ABST) (38). In our study, participants were asked about the frequency of ABST recommendations provided in the past year. Surprisingly, both para-veterinarians and veterinarians reported advising ABST only a few times, raising significant concerns (Figure 9). Similar findings have been documented in other studies, highlighting the low prevalence of ABST in their respective areas (29, 39). This raises concerns because empirical antibiotic prescriptions can lead to treatment failures, prolonged treatment durations, and ultimately reduced antibiotic efficacy in animals (40).

One possible reason behind low ABST rates may be the geographical distance between their practicing area and the testing laboratories. Notably, more than half of veterinarians and para-veterinarians indicated that testing centres are located more than 20 kilometres away from their practicing area. Another factor contributing to the infrequent ABST recommendations is the turnaround time for receiving test results, with most respondents selecting 1–3 or 4–5 days (Table 3). Although 1–3 days represents the standard duration for conducting antimicrobial susceptibility tests, a longer timeframe may be perceived as service delays. Establishing closer testing centres and rapid resting kits can promote ABST use, leading to more targeted therapy and reduced antibiotic resistance, benefiting animal health and veterinary care efficacy.

In cases of treatment failure, most participants opt to repeat the antibiotic. However, a significant difference arises in their approaches. Many para-veterinarians directly change the antibiotic without initially altering the dose or route, while less than half of veterinarians choose this option (Figure 8). A similar pattern was observed in a study conducted in Haryana, India, where most para-veterinarians did not opt for alternative routes and instead indiscriminately used antibiotics (28). An alarming study conducted by Chung et al. highlighted that rapid switching the antibiotics during treatment can exacerbate the development of resistance mutations in pathogens (41). To address the issue of frequent switching of antibiotics, it is advisable to incorporate microbiological testing into the decision-making process. If first-line therapy appears ineffective, antibiotic change should be made after careful assessment (37).

In 2017, the World Health Organization (WHO) identified certain antibiotics as “Watch” group antibiotics for human use due to their higher resistance potential (42). Similarly, the World Organization for Animal Health (OIE) has classified certain antibiotics as VCIA (Veterinary Critically Important Antibiotic) for cautious veterinary use (43). In our study, the majority of participants expressed their preference for a specific antibiotic they were comfortable with (Table 3). Notably, Cephalosporins, Tetracyclines, and Penicillins emerged as the most preferred antibiotic classes (Figures 10, 11). Among these classes, Ceftriaxone (a 3^rd^ generation cephalosporin) and Oxytetracycline (a Tetracycline) fall within the “Watch” category group identified by WHO. In contrast, the VCIA category includes Amoxicillin, Enrofloxacin, Gentamicin, ceftriaxone, and oxytetracycline. Strikingly, these antibiotics are among the top preferences of the respondents. Findings from previous studies have also suggested that veterinarians are prescribing critically important antibiotics as their prime preferences (44, 45). Such frequent use of these critically important antibiotics may contribute to the exacerbation of AMR.

Furthermore, there is a concerning aspect regarding the use of these antibiotics as they come into contact with animals and humans, increasing the risk of human exposure and resistance development in pathogens (46). The reason behind preferring broad-spectrum antibiotics could be multifaceted but given these preferences, the situation appears alarming because, instead of prescribing these critical antibiotics in low frequency, the respondents have made them their top choices. Embracing a One Health approach is crucial to address this issue, fostering collaboration among veterinarians, environmental experts, and healthcare professionals. This approach promotes responsible antibiotic practices, mitigates resistance development, and safeguards public health.

## 5 Limitations of the study

While efforts were made to engage many veterinary professionals, the limited number of participants may pose a constraint on the generalizability of the results. Nevertheless, the narrow 95% confidence interval for the responses enhances the confidence in our findings. It is acknowledged that an in-depth exploration of this domain may not offer a comprehensive overview of the country’s Antimicrobial Resistance (AMR) situation. However, the outcomes will offer insights into the gaps and scenarios prevalent at the base level.

## 6 Ethical clearance

The research received formal ethical approval from the Institutional Human Ethics Committee of the CSIR-IGIB, New Delhi [proposal 17-A, CSIR-IGIB/IHEC/2021–22/02] which authorized the study’s conduct.

## 7 Conclusion

The study illuminates potential behavior and knowledge gaps that are contributing to the AMR in livestock. Notably, para-veterinarians exhibit areas of limited awareness regarding the judicious use of antibiotics, often relying more on colleagues than authentic sources, indicating a need for comprehensive training. A key deficiency identified is the lack of guidance provided by veterinarians to farmers concerning antibiotic withdrawal periods. Addressing these communication gaps through enhanced outreach and guidance from veterinarians is crucial. Notably, prescription practices differ, with para-veterinarians aiming for 100% recovery, while veterinarians prioritize evidence of infection over full recovery.

Both professional groups commonly neglect to advise lab culture tests before prescribing antibiotics to animals. Despite the challenges posed by limited diagnostic infrastructure, especially for labs located over long distances away, educating farmers about the importance of susceptibility tests becomes crucial. However, only a small percentage of veterinarians and para-veterinarians adhere to this practice, reflecting a need for greater awareness. One alarming trend observed is the widespread preference for WATCH and VCIA category drugs by practitioners, potentially depleting antibiotic reserves rapidly.

In light of these findings in Jhunjhunu district collective initiatives by policymakers, practitioners, and farmers are needed. Practitioners must develop a habit of informing animal owners about crucial antibiotic-related information, such as adherence to withdrawal periods. Additionally, Comprehensive training programs, improved access to diagnostic facilities, and regular evaluations of veterinary professionals’ knowledge and skills are essential for promoting responsible prescription practices.

## Data availability statement

The original contributions presented in the study are included in the article/Supplementary material, further inquiries can be directed to the corresponding author.

## Ethics statement

The studies involving humans were approved by the Institutional Human Ethics Committee of the CSIR-IGIB, New Delhi [proposal 17-A, CSIR-IGIB/IHEC/2021-22/02]. The studies were conducted in accordance with the local legislation and institutional requirements. The participants provided their written informed consent to participate in this study.

## Author contributions

VD: Data curation, Formal analysis, Methodology, Writing – review & editing. BR: Data curation, Formal analysis, Methodology, Writing – original draft. AK: Conceptualization, Data curation, Methodology, Project administration, Validation, Writing – review & editing. VS: Conceptualization, Data curation, Methodology, Project administration, Supervision, Writing – review & editing.
