# Supplementary material for: Accessing the behavior and awareness of veterinary professionals towards antimicrobials use and antimicrobial resistance in Indian district
Source: Front Vet Sci. 2024 Mar 11;11:1342089. doi: 10.3389/fvets.2024.1342089 (PMC10962261; doi:10.3389/fvets.2024.1342089)
Supplement: Supplementary file 1 [file Data_Sheet_1.docx]

**Annexure-** Questionnaire pattern

**SECTION – I**

Q1. Name _____________

Q2. E-mail _____________

Q3. Gender

- Male
- Female

Q4. Age

- Below 30
- Between 31-40
- Between 41-50
- Greater than 50

Q5. Experience (in years)

- Less than 5
- Between 5-10
- Between 10-20
- More than 20

Q6. Highest qualification

- BVSc & AH
- MVSc & AH
- Diploma in livestock and animal husbandry
- Other diploma
- Other

Q7. Working sector

- Government sector
- Private sector
- Other

**SECTION – II**

Q8. Overuse of antibiotics in animals is responsible for antibiotic resistance?

- Agree
- Disagree
- Agreed to some extent
- Don't agree, Don't disagree

Q9. Improper use of antibiotics contributes to antimicrobial resistance?

- Agree
- Disagree
- Neither agree nor disagree
- Other

Q10. There has been an increase in the number of cases of antimicrobial resistance in your work area

- Agree
- Disagree
- Neither agree nor disagree

**SECTION – III**

Q11. What makes you prescribe antibiotics?

- Evidence of infection
- To ensure 100% recovery
- Lab culture test
- Other

Q12. Dose calculation would be based on?

- Body weight
- By experience
- By guess
- Always use a whole vial

Q13. Do you inform cattle farmers, about the used antibiotic and its withdrawal period?

- Yes
- Rarely
- Maybe

Q14. When do you repeat the antibiotic dose?

- Only when there is improvement
- If the owner calls
- Other

Q15. What do you generally prescribe during the repeat dose of antibiotic?

- Antibiotic change
- Higher dose of previously used antibiotic
- Dose route change
- Other

Q16. In the last one year, how many times have you advised and asked for an antibiotic sensitivity test?

- 1-5 times
- >20 times
- 6-10 times
- 11-15 times
- 16-20 times

Q17. How do you measure the body weight of an animal?

- Experience
- Idea
- Other

Q18. Are you relying on laboratory results before prescribing an antibiotic?

- Yes
- No
- Maybe

Q19. Do you read the label of the antibiotic before use?

- Yes
- Rarely

Q20. Approx distance (in KMs) of any laboratory, that has the facility of antibiotic sensitivity from your practicing area?

- 1-3 Km
- 4-7 Km
- 7-10 Km
- 10-15 Km
- 15-20 Km
- >20 Km

Q21. Approx turn around time for the antibiotic sensitivity test results?

- 1-3 days
- 4-5 days
- 5-7 days
- 8-10 days
- >10 days

Q22. Is there any antibiotic that you feel comfortable prescribing?

- Yes
- No

Q23. Name of the First/Second and Third preferred antibiotics?

- Aminoglycosides
- Amoxicillin
- Amoxycillin + cloxacillin
- Ampicillin
- Cefalexin
- Ceftiofur
- Ceftriaxone
- Ceftriaxone + sulbactum
- Cephalosporins
- Chloramphenicol
- Ciprofloxacin
- Cloxacillin
- Colistin
- Dexamethansone
- Enrofloxacin
- Erythromycin
- Fluoroquinolones
- Fosomycin
- Gentamicin
- Metronidazole
- Neomycin
- Norfloxacin
- Ofloxacin
- Oxytetracycline
- Penicillin
- Streptomycin
- Streptomycin and Penicillin
- Streptopencillin
- Sulbactam
- Sulfachloropyrazine
- Sulfadiazine
- Sulfadimidine
- Sulpha drugs
- Sulfamethoxazole
- Tazobactam
- Tetracycline
- Tetracycline group
- Trimethoprim
- Tylosin
- Other
